# Supplementary material for: Delivery, setting and outcomes of paediatric Outpatient Parenteral Antimicrobial Therapy (OPAT): a scoping review
Source: BMJ Open. 2018 Nov 15;8(11):e021603. doi: 10.1136/bmjopen-2018-021603 (PMC6252693; doi:10.1136/bmjopen-2018-021603)
Supplement: Supplementary file 1 [file bmjopen-2018-021603supp001.pdf]

Author, Year Country	Design, Sample size, Age of children	Underlying condition/ Indication for treatment	Key requirements /influences on offering OPAT/inclusion & exclusion criteria	Setting (DTC/Home)/ Delivery	Key clinical outcomes/adverse events (Note: for some papers events categorised as adverse by scoping reviewers)	Other outcomes (e.g., finance, family perspectives)	Author-noted limitations	Author-noted comments/ recommendations
Banerjee et al. (2014)<br><br>USA	Online survey of paediatric physicians (survey open for 2 months)<br><br>158 physicians<br><br>Child's age = NA	**Underlying condition**<br>• Various but not reported.<br>**Indication for treatment**<br>• Most commonly osteomyelitis, endovascular central nervous system infections, pneumonia	**Inclusion criteria**<br>• Patient compliance, home environment,<br>• Need for parenteral medication<br>• Resources for follow-up/ monitoring<br>• Patient age<br>• Presence of bacteraemia	**Setting**<br>• Not reported<br>**Delivery**<br>• Most (94%) reported that their institution had a team for peripherally inserted central catheter placement.	**Adverse events**<br>• 104 (67%) respondents reported that line or drug-associated complications occurred in 10% or fewer cases.	**Financial**<br>• Revenue sources reported to support OPAT management included: outpatient visit charges (51%), inpatient consult charges (36%), support from hospital or healthcare system (18%), support from home care agency (12%), or income from infusion center (4%).<br>• 32% of respondents did not know the revenue source for OPAT management and 5% reported OPAT services were not financially supported.	• Responses regarding OPAT programs and clinical practice, may not represent actual practice.<br>• Responses may be subject to recall bias.<br>• The generalizability of the findings to all pediatric ID practices is uncertain.<br>• No assessment of the extent to which OPAT use varied by the presence of comorbidities.<br>• Survey did not collect data on all of the clinical and social factors that contribute to decision making regarding OPAT use.	• OPAT use in paediatrics is common.<br>• Most respondents significantly underestimated the risk of OPAT-related complications.<br>• Substantial variation in the characteristics of pOPAT practices and frequency of OPAT use.<br>• Many pOPAT programs may lack adequate resources and infrastructure to perform appropriate follow-up of OPAT patients.<br>• Better evidence is needed regarding not only the benefits of pOPAT.<br>• Decisions to use OPAT are complex, and reasons for the variability in OPAT use by pediatric ID providers should be further explored.
Campo et al. (2001)<br><br>Spain	Retrospective review (6 years)<br><br>7 children receiving home parenteral nutrition (and 11 adults, whose data are not reported here).<br><br>Mean age 3.1, Range 6 months-8 year.	**Underlying condition**<br>• Intractable diarrhoea, short bowel, motility, liver cirrhosis.<br>**Indication for treatment**<br>• Catheter-related infection	**Inclusion criteria**<br>• Existing CVC for infusion of HPN<br>• All the HPN patients/ families/ caregivers competent in handling CVC and avoiding and recognising complications.	**Setting**<br>• Home<br>**Delivery**<br>• Infusion performed after completion of HPN.<br>• If the patient's general condition was stable, the whole therapy could be received at home. Otherwise, the patient remained in hospital until complete remission of fever occurred and sensitivities were known.	**Key clinical outcomes**<br>• No differences in the length of antibiotic treatment in CRBSI between patients admitted to the hospital or treated entirely at home.<br>**Adverse events**<br>• Incidence rate of CR-BSI was 0.9 in children.<br>• Incidence rate of tunnel infection was none in children.<br>• 4 children never developed CR-BSI. Three children with episodes of CR-BSI were treated as inpatients.<br>• There were no adverse effects during OPIVAT	**Financial**<br>• The National Health System in Spain covers all the expenses due to IV nutrition both in the inpatient and the outpatient setting, including those due to the complications of the technique.	• None reported.	• Use of OPIVAT requires adequate selection criteria and patient evaluation. Patients must be medically stable, the home environment should be clean, have a telephone, etc.<br>• Children develop CR-BSI more frequently than adults<br>• When a catheter infection occurs antibiotic parenteral therapy may be administered totally or partially at home. Cost-savings and benefits for the patient may be significant.
Cantero (2014)<br><br>Spain	Prospective descriptive.<br><br>55 children (33 males and 22 females). 35 patients (63.6%) had more than one episode = total of 163 episodes of home parenteral treatment<br><br>Mean age 11.1 years	**Underlying condition**<br>• Cystic fibrosis, cancer, gastrointestinal diseases, HIV infection, bronchopulmonary dysplasia and hyper IgM syndrome.<br>**Indication for treatment**<br>• The main sources of the treated infections were respiratory tract (76%), catheter-related bloodstream (9.2%), and urinary tract infections (5.5%).	**Inclusion criteria:**<br>• Medically stable patient (assessed by interview by HHU team),<br>• chronic underlying disease,<br>• non-progressing infection,<br>• family support, carers physically & mentally able to provide treatment in the home,<br>• home address within the HHU service area, has a telephone, running water, and refrigerator,<br>• voluntary consent for at-home treatment, signed informed consent<br>**Exclusion criteria:**<br>• Infection can be treated orally or requires other inpatient treatment	**Setting**<br>• Home<br>**Delivery**:<br>• Long-term patients: HHU nurses train long term patient and the carer (in hospital) to feel to self-administer the medication and evaluate potential complications.<br>• Shorter treatments: HHU nursing staff perform home visits to administer the treatment (the patient and carers trained to assess for complications).<br>• Daily phone call from HHU team while patient is home and treatment is ongoing; home visits from member of HHU team as needed.	**Key clinical outcomes**<br>• Most treatments (96.6%) were IV; IM route in 5 (3%) episodes.<br>• Peripheral access (94.5%); central access only used in 4 (2.5%) patients who had a CVC.<br>• 147 episodes (90.2%) of treatment at home prior to discharge.<br>• Mean duration of home treatment 11.05 days (SD 5.82, range 1-25 days)<br>• Cumulative number of treatment days was 1972<br>**Adverse events**<br>• Extravasation or accidental displacement of the peripheral line (n=12, 7.2%), phlebitis (n=5, 3%), exanthema or urticaria concurrent with drug administration (n=5, 3%), poor technique in antimicrobial administration due to incorrect flushing of the device (n=1, 0.6%), and breakdown of the infusion pump (n=1, 0.6%).<br>• 16 patients (9.8%) episodes required admission to the hospital; 4 (2.5%) to poor evolution of the infectious process despite treatment, and 12 (7.4%) due to an exacerbation of the underlying disease.<br>• No deaths occurred.	**Financial**<br>• An estimated cost-analysis found that the inclusion of patients in this programme could have saved up to 95% of the cost of conventional inpatient treatment	• It is a single-centre study<br>• The majority of referrals were CF patients.	• Essential factors for a successful OPAT programme are: appropriate infrastructure, careful selection of patients, multidisciplinary staff with experience both in at-home care delivery and in the diagnosis and treatment of infectious diseases, provision of information and training, good monitoring of the patient, guaranteed 24 h a day by hospital to address any complications that may arise.

Author, Year Country	Design, Sample size, Age of children	Underlying condition/ Indication for treatment	Key requirements /influences on offering OPAT/inclusion & exclusion criteria	Setting (DTC/Home)/ Delivery	Key clinical outcomes/adverse events (Note: for some papers events categorised as adverse by scoping reviewers)	Other outcomes (e.g., finance, family perspectives)	Author-noted limitations	Author-noted comments/ recommendations
Doré-Bergeron et al. (2009)<br><br>Canada	A retrospective cohort study using record review (20 months)<br><br>66 days (range: 33– 85 days).<br><br>118 infants; 67 (56.8%) treated in day treatment centre (DTC), 51 (43.2%) hospitalized.	**Underlying condition**<br>• No other conditions reported<br>**Indication for treatment**<br>• Urinary tract infections.<br>• Diagnosis of urinary tract infection was confirmed for 86.6% of DTC patients<br>• Escherichia coli was identified in 84.5% of urine cultures; 98.3% of isolates were sensitive to gentamicin. Six blood cultures (10.3%) yielded positive results, 5 of them for E coli.	**Inclusion criteria:**<br>• Children who appeared nontoxic, who had normal renal function, and who met no other exclusion criteria<br>**Exclusion criteria:**<br>• age of 30 days,<br>• toxic appearance or dehydration,<br>• abnormal renal function,<br>• dubious parental compliance,<br>• history of urinary tract surgery,<br>• abnormal cerebrospinal fluid (CSF) findings (leukocyte count of 10 cells per L or protein level of 0.40 g/L),<br>• other serious medical conditions.	**Setting:**<br>• DTC<br>**Delivery**<br>• All infants were initially assessed by ED pediatricians.<br>• The infants were monitored on a daily basis by DTC staff until symptoms improved and culture results were obtained.<br>• Daily visits by paediatric nurses trained in the delivery of parenteral antibiotic therapy on an ambulatory basis until the end of IV treatment<br>• Antibiotic administered via peripheral IV.<br>• Parents were asked to measure the child's rectal temperature every 4 hours at home during intravenous treatment<br>• More than one third of patients (36.4%) lived 20 km from the hospital.	**Key clinical outcomes**<br>• Treatment with IV antibiotics in DTC lasted a mean of 2.7 days.<br>• 86.2% of patients with confirmed UITs were successfully treated in DTC (defined as attendance at all visits, normalization of temperature within 48 hours, negative control urine and blood culture results, if cultures were performed, and absence of hospitalization from DTC).<br>• Overall success of treatment was lower for young infants, but this result was not statistically significant.<br>**Adverse events**<br>• 7 patients were hospitalized from the DTC (n=1, severe concomitant gastroesophageal reflux; n=1 right hydronephrosis). Five of 6 children with bacteraemia were hospitalized because of the positive blood culture results. N=1 with bacteraemia who was not hospitalized also had an uneventful course.<br>• Minor problems with intravenous access, including failure to establish IV access (gentamicin was administered intramuscularly) and the need to replace the IV line during the course of therapy, encountered for 5 patients (8.6%).<br>• Seven of the 8 "treatment failures" were considered failures because of hospital admission	**Parental**<br>• Rate of parental compliance with DTC visits was 98.3%.	• Long-term results (e.g., rate and extent of renal scarring) for DTC patients not measured.<br>• Results might have been different in another context.	• Ambulatory treatment of infants 30 to 90 days of age with febrile UTIs using short-term, IV antibiotic therapy at a DTC is feasible.<br>• This approach may reduce significantly the number of hospital admissions for UTI treatment in this age group.<br>• Excellent parental compliance can be achieved in a DTC, which allows close medical supervision
Gauthier et al. (2004)<br><br>Canada	Prospective, observational (1 year).<br><br>Parental satisfaction survey.<br><br>291 episodes of presumed febrile UTI were diagnosed in the ED, of which 212 (72.9%) were sent to the DTC<br><br>Patients 3 months to 5 years of age. Patients treated at the DTC, with a final diagnosis of UTI, had a median age of 12.0 months (range: 3-68 months),	**Underlying condition**<br>• No other conditions reported<br>**Indication for treatment**<br>• Presumed febrile UTI<br>• Children were considered febrile if their parents reported fever at home or if, in the ED, they had a body temperature of 38.5°C rectally or 38°C orally.	**Exclusion criteria**:<br>• Toxic appearance,<br>• 5% dehydration,<br>• dubious parental compliance,<br>• parental inability to comply with the physical requirements of treatment at the DTC or parental refusal,<br>• known significant uropathy,<br>• history of surgical intervention involving the urinary tract during the past 3 months,<br>• hearing deficit,<br>• abnormal serum creatinine levels,<br>• other serious medical conditions.	**Setting:**<br>• DTC<br>**Delivery**<br>• Evaluation performed in the ED included a complete blood count, blood culture, measurement of serum blood urea nitrogen, creatinine, and electrolyte levels, urinalysis, and urine culture.<br>• IV antibiotics administered via peripheral line.<br>• Parents were given written information about follow-up care at the DTC	**Key clinical outcomes**<br>• Duration of IV antibiotic therapy at the DTC was 1.9 days (SD: 0.9 day).<br>• Mean number of visits to the DTC, including appointments for renal US and voiding cystourethrography evaluations, was 3.5 (SD: 0.9).<br>• Parents were present at all scheduled visits in 98.9% of cases.<br>• Parents refused referral to the DTC or were judged unable to comply with DTC treatment by ED physicians in only 9 instances; so children hospitalized.<br>• Patients afebrile by 24 hours in 52% of UTI episodes and by 48 hours in 82%.<br>• At telephone follow-up assessments 14 days after discharge, no patient had been rehospitalized because of UTI.<br>• Successful treatment at the DTC (defined as attendance at all visits, normalization of temperature within 96 hours, negative control urine cultures, if performed, and absence of hospitalization from the DTC) observed in 96.6% of the 178 UTI episodes.<br>**Adverse events**<br>• Minor problems with IV access occurred in 9.0% of cases.<br>• Four patients hospitalized from the DTC, only 1 case related to UTI treatment.<br>• Problems with IV access occurred in 16 instances (9.0%) (e.g., failure to establish IV access, need to reinstall IV access).during the course of therapy. No major IV complications reported	**Financial**<br>• The average daily cost for hospitalization on the wards where children with UTI are usually admitted estimated at CAN $400 (US $300) vs daily cost for treatment at the DTC was CAN $52 (US $39); these rates did not include medication, investigation costs, and physician fees, which would be approximately the same in both settings.<br>**Parental**<br>• 172 anonymous parental satisfaction questionnaires returned: 75% described the overall experience at the DTC as excellent, 20.3% as very good, and 3.5% as good. Only 2 parents (1.2%) considered their experience fair or poor. Of 43 parents whose child had been hospitalized in the past, 65.1% thought that their experience at the DTC was much better than their hospital experience, 20.9% that it was better, and 9.3% that it was similar. 32% and 20.4% expressed worry at the thought of going home with a child who was still febrile and who had indwelling IV access, respectively	• None reported.	• Ambulatory treatment with IV antibiotics, at a DTC, may be used for at least three-fourths of UTIs among febrile children 3 months to 5 years of age.<br>• DTC treatment is safe and feasible and appears very satisfactory to parents.

Author, Year Country	Design, Sample size, Age of children	Underlying condition/ Indication for treatment	Key requirements /influences on offering OPAT/inclusion & exclusion criteria	Setting (DTC/Home)/ Delivery	Key clinical outcomes/adverse events (Note: for some papers events categorised as adverse by scoping reviewers)	Other outcomes (e.g., finance, family perspectives)	Author-noted limitations	Author-noted comments/ recommendations
Glackin et al (2014)<br><br>Ireland	Retrospective record review (3 years)<br><br>32 children who received a total of 361 OPAT courses.<br><br>The median age was 8.8 years (range 2.75- 17.8 years). Sixteen (50%) were male	**Underlying condition**<br>• Cystic fibrosis (n=30), recurrent pneumonia (n=2).<br>**Indication for treatment**<br>• All children treated with OPAT had pneumonia.<br>• Common organisms included Pseudomonas aeruginosa (mucoid and non-mucoid), Staph aureus (methicillin sensitive or methicillin resistant), Haemophilus influenza, Streptococcus pneumonia or a combination.	**Inclusion criteria**<br>• Usual practice stated to be individual assessment by consultant respiratory paediatrician and CF nurse specialist for suitability for home IV antibiotics	**Setting**<br>• Home<br>**Delivery**<br>• The CF nurse specialists train parents (administration, storage of medications, hygiene, IV access care, monitoring for all potential side effects, plan of action in the event of same including names and contact numbers at the hospital.<br>• Parent training time to reach competency in OPAT administration etc. was 3-5 days, while re-training was usually < 1 day.<br>• All patients had 24hr access to medical assistance either directly or over the phone with the respiratory team or via the in-house medical registrar.	**Key clinical outcomes**<br>• 3,688 days of intravenous antimicrobials administered at home using the OPAT programme.<br>• On average, children had 11 courses (range 2-112) over the 3 year period, with a mean duration of 10 days therapy (range 2-42 days).<br>• 23 (72%) of children treated with OPAT had portacaths, the remainder used long lines sited by the hospital IV team.<br>• For children on the active lung transplant list, some courses were extended to 4-6 weeks with a change in antibiotic choice after 2-3 weeks.<br>**Adverse events**<br>• 3 (2%) portacath infections. All three were surgically removed after failure to respond to antimicrobial therapy while awaiting surgery.<br>• One (0.6%) re-admission: a child who had a deterioration in pulmonary status and chest radiograph findings.	• None reported	• None reported	• Patient benefits include reduced risk of health care associated infections and higher levels of satisfaction with OPAT (in appropriate conditions) than with inpatient hospital care<br>• Success of OPAT is dependent on appropriate patient selection, weekly follow up of patient clinical status, blood tests, 24 hour access to medical advice and overall adherence to national practice guidelines.<br>• CF is suited to OPAT because chronicity of condition and need for repeated courses of antimicrobials parents become trained and experienced, children and families are well known to the respiratory team and many of these children have permanent indwelling IV access (portacaths) in place.<br>• In current climate of budget cuts, pressure on inpatient bed availability and risk of nosocomial infections, OPAT is an important and effective tool
Goldman et al. (2017)<br><br>USA	Record review of the Truven MarketScan Medicaid claims database (4 years)<br><br>Paediatric Medicaid enrollees (aged 0-18 years).<br><br>3433 distinct OPAT episodes for 2687 patients.	**Underlying condition**<br>• No specific conditions reported<br>**Indication for treatment**<br>• Infections related to haematology/ oncology, gastrointestinal/ genitourinary cystic fibrosis, osteoarticular, and pulmonary, bacteraemia, vascular/endocarditis, upper respiratory infection, soft tissue infection, central nervous system, urinary tract infection, other.	• Not reported	**Setting**<br>• Not reported	**Adverse events**<br>• More than one third of children receiving OPAT (n = 1289; 38%) had either an ED visit or hospitalization during an OPAT episode.<br>• Haematology/oncology diagnostic category was associated with the highest percentage of medical care encounters with 28% experiencing an ED visit and 51% having a hospitalization during an OPAT episode.<br>• Other categories with high rates of healthcare encounters were endovascular/endocarditis (27% ED, 24% hospitalization) and GI/GU (24% ED, 30% hospitalization).<br>• Overall, 61% of acute healthcare encounters during OPAT episodes were likely attributable to a catheter-related complication; this rate was relatively consistent across diagnostic categories<br>• Among children who experienced an OPAT-related complication, 25% were treated with a highly bioavailable antimicrobial.<br>• Of the 791 episodes of OPAT-related inpatient or ED use, 265 (33%) included ICD-9 code for fever, 276 (35%) included ICD-9 code for line complication, and 250 (32%) included ICD-9 codes for both fever and line complication over 20% of OPAT episodes resulted in children requiring medical care in the hospital or ED setting for an OPAT complication	• None reported	• Findings may not be generalizable to other US regions or commercially insured children.<br>• Unable to determine the population-based incidence of OPAT as database does not provide the total population of Medicaid enrollees.<br>• A conservative approach to defining OPAT may have resulted in an underestimation of the number of children prescribed OPAT.<br>• Lack of chart review may have resulted in misclassification of indication for OPAT, reasons for subsequent healthcare use, and appropriateness of OPAT.<br>• An overestimation of the complication rate by including fever may be reason for high OPAT complication rate (ED visit or hospitalization)	• OPAT is used for children with a wide spectrum of clinical diagnoses and for the administration of a wide variety of antimicrobial agents, including antibacterials and antifungals.<br>• A substantial number of OPAT episodes included highly bioavailable antimicrobials prescribed intravenously that could potentially have been administered orally.<br>• Patients receiving OPAT are at high risk for requiring additional ED and inpatient hospitalizations during their OPAT episode, and the majority of these healthcare encounters were likely related to OPAT complications<br>• Failure to switch from OPAT to oral administration when using highly bioavailable agents can result in higher medical care cost and the potential for harm without evidence of therapeutic benefit<br>• The high cost and potential for complications means additional studies are needed to compare the effectiveness of OPAT to oral therapy for other conditions as well as comparisons between longer and shorter durations of IV therapy<br>• The integration of stewardship principles into clinical decision making prior to OPAT initiation is critical.

Author, Year Country	Design, Sample size, Age of children	Underlying condition/ Indication for treatment	Key requirements /influences on offering OPAT/inclusion & exclusion criteria	Setting (DTC/Home)/ Delivery	Key clinical outcomes/adverse events (Note: for some papers events categorised as adverse by scoping reviewers)	Other outcomes (e.g., finance, family perspectives)	Author-noted limitations	Author-noted comments/ recommendations
Gomez et al. (2001)<br><br>USA	Retrospective record review (4.5 years)<br><br>229 patients received 237 courses of OPAT<br><br>Mean age of 8 years (median, 8 years; range, 14 days to 19 years).<br><br>Majority of patients were male [127 (55%)] and Caucasian [170 (74%)].	**Underlying condition**<br>• No specific conditions reported<br>**Indication for treatment**<br>• Many different types of infections were treated with OPAT, musculoskeletal infections remained the most commonly treated infection.	• Not reported.	**Setting**<br>• Home<br>**Delivery**<br>• Since study was retrospective, medical & social criteria used in patient evaluation for OPAT could not be reviewed.<br>• However, usual practice reported as follows: prior to discharge, family and the home situation evaluated and at least 2 family members trained (administration, catheter management, catheter-associated complications and adverse drug reactions).<br>• Usual practice, initial daily nurse visits, then at least once a week while OPAT was administered. And patients seen at least once every week or every other week, and weekly laboratory tests were done	**Key clinical outcomes**<br>• Primary IV access included central venous catheters for 125 (53%) courses, peripherally inserted central catheters for 99 (42%), peripheral IV catheters for 7 (3%) and unknown for 6 (2%) courses. The use of peripherally inserted central catheters increased from 33% in 1995 to 59% in 1998.<br>• During the review period: OPAT use increased by almost 4-fold; patients <=2 years of age, showing a 7-fold increase;<br>• 42 (18%) patients <=1 year of age and 1 (0.4%) <=1 month of age received OPAT during the study period.<br>• Average post-OPAT follow-up period was 8 weeks (range, 1 to 67 weeks). The mean duration of therapy (31 days)<br>**Adverse events**<br>• Average time to developing catheter-associated complications was 22 days.<br>• Catheter-associated complications (CAC) prompted cessation of OPAT in 17 (7%) courses.<br>• Adverse drug reactions (ADRs) developed in 70 (29%) OPAT courses and prompted early discontinuation of antibiotics in 58 (24%). Both ADRs and CACs developed in 20 (8%) OPAT courses.<br>• Average time to ADRs was 19 days (range 1–71 days).<br>• Rehospitalization was necessary for 62 (26%) OPAT courses. CACs and catheter changes prompted rehospitalization in 26 (42%) courses, ADRs in 17 (27%), surgery for treatment of primary disease in 18 (29%) and various other causes in 5 (8%). A few patients were rehospitalized more than once or had more than one reason for rehospitalization.	• None reported	• Quality of life issues during OPAT could not be addressed in this retrospective analysis.	• Further studies are needed to gain insight into the impact of OPAT on patients' and families' lives.<br>• Close monitoring of potential complications is needed.<br>• Medical and social criteria must apply when identifying suitable candidates.<br>• Paediatric guidelines for OPAT similar to adult guidelines need to be established.
Gutpa et al. (2009)<br><br>INDIA	Single institutional, randomized control trial of oncology children with low-risk febrile neutropenia (LRFN)<br><br>88 patients (67 males, 21 females) with 123 episodes of OPAT (62 randomised to oral arm; 61 to IV arm). Median age was 8.25 years (oral) and 7.75 years (IV)	**Underlying condition**<br>• Acute lymphoblastic leukaemia, primitive neuro-ectodermal tumour, rhabdomyosarcoma and osteosarcoma<br>**Indication for treatment**<br>• Low-risk febrile neutropenia (LRFN)	**Inclusion criteria**<br>• patients with age 2-15 years; absolute neutrophil count (ANC) r500/mL;<br>• normotensive;<br>• no clinical evidence of lower respiratory tract infection;<br>• normal chest radiograph;<br>• presence of reliable caretakers;<br>• availability of telephone contact<br>• residence less than 1 hour from the medical centre.<br>**Exclusion criteria**<br>• Conditions normally requiring hospitalization (e.g., such as dehydration, severe mucositis, pneumonia, typhlitis);<br>• intensive leukaemia/ lymphoma treatment except maintenance therapy in acute lymphoblastic leukaemia;<br>• stem cell transplantation;<br>• refractory malignancy;<br>• renal insufficiency;<br>• severe biochemical derangements;<br>• hepatic dysfunction;<br>• neutropenia predicted to last more than 10 days after onset of fever;<br>• past history of invasive fungal infections	**Setting:**<br>• Outpatient clinic of study hospital or nearby medical clinics.<br>**Delivery**<br>• Patients were instructed to record their temperature at home 3 times daily and bring the temperature charts to the clinic every 24 to 48 hours or as often as indicated by the clinical condition where they were assessed clinically and complete blood count was checked.<br>• If unable to come at 24 hours, a phone call was made to ensure the stability of the patient.	**Key clinical outcomes**<br>• There were 27.1% episodes in the oral arm and 24.1% episodes in the intravenous arm with no documented focus of infection.<br>• Treatment of FN was successful in 55/61 (90.16%) episodes in oral arm and 54/58 (93.10%) in IV arm.<br>**Adverse events**<br>• 3 hospitalizations (all in the oral arm; seizures, bleeding and nonresolution of fever in one patient each) and no mortality.<br>• In the 4 patients excluded from analysis there was no mortality and all underwent successful therapy of their episodes of FN. Three of the 4 excluded patients had inadvertently received cefoperazone-sulbactam instead of ceftriaxone.<br>• There were 6 failures in oral and 4 failures in the intravenous arms. Subgroup analysis revealed that failure was associated with diarrhoea in the IV arm and use of oral therapy in RMS patients receiving VAC.	• None reported	• Study was underpowered to draw conclusions about mortality (extremely low-risk/low-incidence event in this group of patients)	• Careful selection of patients is important: patients with controlled disease,<br>• clinically well, reliable caretakers and whose home was less than 1-hour journey from the hospital<br>• Maintaining daily telephone contact with the patient. helps in monitoring patient progress when patients are unable to attend clinic daily, in times of emergency and also helps to ensure patient compliance.<br>• The risk factors significantly associated with failure in our study could help in refining criteria for pediatric LRFN and planning further studies.

Author, Year Country	Design, Sample size, Age of children	Underlying condition/ Indication for treatment	Key requirements /influences on offering OPAT/inclusion & exclusion criteria	Setting (DTC/Home)/ Delivery	Key clinical outcomes/adverse events (Note: for some papers events categorised as adverse by scoping reviewers)	Other outcomes (e.g., finance, family perspectives)	Author-noted limitations	Author-noted comments/ recommendations
Hodgson et al. (2016)<br><br>Australia	Prospective, observational (12 months).<br><br>228 patients received OPAT in 251 episodes.<br><br>Median age: 7.4 years (range 1 week to 21 years); 22 patients (10%) under 1 year	**Underlying condition**<br>• Most patient referrals from inpatient wards; general medicine (55, 24%), haematology/ oncology (38, 17%), respiratory (29, 13%) orthopaedics (25, 11%)<br>• 42(18%) were referred directly from the emergency department (ED).<br>**Indication for treatment**<br>• Most frequent diagnoses for which patients received OPAT were exacerbation of cystic fibrosis and urinary tract infection (UTI).<br>• Of the 42 admitted directly from the ED, the majority were for skin and soft-tissue infection (25, 60%) and UTI (14, 33%). The remaining few were pneumonia worsening despite oral antibiotics, CLABSI and septic arthritis	•	**Setting**<br>• Home<br>**Delivery**<br>• All antibiotics were administered by trained nurses, not parents.<br>• Central venous catheter (CVC) care was consistent with hospital guidelines: no-touch sterile technique, dressings changed every seven days and inspection daily to identify insertion-site infection.<br>• Antimicrobial agents were administered either through 24 h elastomeric infusers as a short push or using pumps over a defined time frame.<br>• Visits by HITH nurses occurred daily or twice daily depending on the antibiotic prescribed.	**Key clinical outcomes**<br>• In total, 309 parenteral antibiotics prescribed: 304 IV, 3 IM and 2 IV followed by IM. Simultaneously, 57 patients also had an oral antibiotic and 1 an intranasal antibiotic prescribed.<br>• Overall, only 1 of 251 OPAT episodes was abandoned due to persistently positive blood cultures. All others had resolution of infection by the end of the OPAT course or improvement sufficient to switch to oral antibiotics to complete the course.<br>• Venous access was most commonly peripherally inserted central catheter (29%) and peripheral cannula (29%).<br>• Median length on OPAT was 7 days (range 1–190 days), varied between conditions.<br>• Shortest lengths of OPAT (median of 1–2 days) were for skin abscess, cellulitis, community-acquired pneumonia and UTI.<br>• Longest lengths of stay (median >21 days) were for ascending cholangitis, pyomyositis, chronic osteomyelitis, discitis and cerebral abscess.<br>**Adverse events**<br>• Majority of antibiotics (72%) were prescribed appropriately but 6% deemed an inappropriate choice for the indication and 26% had inappropriate dose or duration<br>• Incidence of central line-associated bloodstream infections was 0.9%.<br>• Unplanned readmission rate was 4%, with low rates of OPAT-related adverse events. Three children (1%) had an inadequate clinical response.<br>• OPAT-related adverse events were all related to vascular access with an overall access complication rate where it was recorded of 11% (14/133): CVC/portacath 4/45 (9%), PICC 4/46 (9%), peripheral cannulae 3/26 (11%) and midline catheters 3/7 (43%).<br>• No antibiotic adverse events necessitating change or cessation of antibiotic or hospital readmission.	**Financial**<br>• Reduction in cost to care for a patient at home receiving OPAT compared with the average cost of care in a hospital bed in study hospital for a medical patient is AU$590/day.<br>• As there were 3084 days where OPAT replaced inpatient care, this represents an estimated cost saving of AU$1.82 million in 1 year.	• This study did not directly compare outcomes of OPAT with inpatient antimicrobial therapy, and these groups may be different.	• In this study population, OPAT appears to be safe (few adverse events), efficacious (low rate of unplanned readmissions) and cost-effective (cheaper than an equivalent inpatient bed stay).<br>• Room for improvement in documentation, drug monitoring and appropriateness and the development and implementation of an OPAT-specific guideline and increased oversight of antimicrobial use will be important.<br>• Depending on clinical response, children can still attend school or nursery, limiting educational interruptions, and parents can therefore attend work<br>• Overall complication rate for PICC, CVC and portacaths combined was 9%, and it is not clear why this rate is so much lower. It may reflect different selection criteria for patients related to high rate of peripheral cannula use, strong hospital education from the Infection Control team about CVC care or possibly under-reporting of complications.
Le et al. (2010)<br><br>USA	Retrospective record review (6 years)<br><br>98 patients (under age 18) who had received care from Memorial Home Health Care Agency	**Underlying condition**<br>• Not reported<br>**Indication for treatment**<br>• osteomyelitis, septic arthritis, pneumonia, cystic fibrosis-related pneumonia, and skin/soft-tissue infections	**Inclusion criteria**<br>• patients should have received OPAT for at least 1 day at home.<br>**Exclusion criteria**<br>• Patients with cancer, sickle cell anaemia and immunodeficiency were excluded	**Setting**<br>• Home<br>**Delivery**<br>• Home health nurses provided instructions on catheter care and antibiotic administration and, in conjunction with clinicians, determined the need for return to the ED or hospital to address any CACs.	**Key clinical outcomes**<br>• Approx. 14% of patients did not have an organism identified, making it difficult to transition to appropriate oral therapy. However, for infections like osteomyelitis and septic arthritis which are usually caused by Gram-positive organisms, an oral antibiotic such as linezolid, though expensive, may be more cost-effective and safer than placing a PICC line and monitoring through a home health agency.<br>**Adverse events**<br>• A total of 36 complications occurred in 32 of 98 patients receiving a course of OPAT.<br>• 25 catheter associated complications (CACs) e.g., Occlusion/clotting (n = 9) and dislodgement (n = 8) and 11 antibiotic associated complications (AACs) e.g. neutropenia (n = 5).<br>• The mean time to development of a complication was 7 days (range 1-28 days) after PICC placement;<br>• The use of OPAT for osteomyelitis was associated with complications (odds ratio = 2.69; 95% confidence interval = 0.99-7.35; P = .05).<br>• Interruption of OPAT from complications resulted in early discontinuation of IV antibiotics.<br>• 17 patients required an unplanned medical care visit to the ED, hospital, or clinic: 15 with CACs and 2 with AACs. 2 were hospitalized to complete a course of IV antibiotics.	• Not reported	• As the standard of care in study hospital was OPAT, there was no control group.<br>• Retrospective design meant could not fully capture the social impact of OPAT, including patients' return to school or parents' return to work.<br>• Unable to conduct long-term follow-up on these patients or determine if patients went to an outside facility for complications	• OPAT therapy was effective, but a third of children on OPAT experienced complications and often required additional health care visits.<br>• Further studies in children with prospective designs are needed to evaluate the social impact of OPAT and the role of alternative approaches to OPAT including the use of oral antibiotics with good bioavailability and activity against MRSA in the outpatient setting.<br>• Evaluation of the costs associated with complications from OPAT is needed and should incorporate visits to the ED and clinics, and rehospitalization. Although OPAT is more cost-effective than hospitalization, it may still be more expensive than oral antibiotic therapy and may not offer any additional benefit.<br>• Given that one third of children experienced complications and half of them required medical attention, alternatives to OPAT should be explored.<br>•

Author, Year Country	Design, Sample size, Age of children	Underlying condition/ Indication for treatment	Key requirements /influences on offering OPAT/inclusion & exclusion criteria	Setting (DTC/Home)/ Delivery	Key clinical outcomes/adverse events (Note: for some papers events categorised as adverse by scoping reviewers)	Other outcomes (e.g., finance, family perspectives)	Author-noted limitations	Author-noted comments/ recommendations
Madigan & Banerjee (2013)<br><br>USA	Retrospective record review (16 months)<br><br>109 unique patients received OPAT; 13 (12%) patients received 2 or more OPAT courses<br><br>The mean patient age was 8.8 years (range: 1 month–20 years), and there was an equal distribution of males (n = 63, 50%) and females (n = 63, 50%).	**Underlying condition**<br>• Not reported<br>**Indication for treatment**<br>• Most common conditions: bone and joint (21%), bloodstream (15%), intra-abdominal (13%) and soft tissue (9%) infections.	**Inclusion criteria**<br>• Providers, together with nurses and pediatric discharge planning specialists assessed stability of the home environment and ability of parents or guardians to administer medications before discharge with OPAT.<br>• The home healthcare nurse provided teaching of parents or guardians before hospital discharge or at the first home visit that generally occurred on the day of discharge. Catheter flushing instructions were dependent on home healthcare agency and catheter type.<br>**Exclusion criteria**<br>• If there were any concerns regarding patient compliance or suitability of the home environment for OPAT, OPAT was generally not used.	**Setting**<br>• Home<br>**Delivery**<br>• Agents were usually infused through pushes, 24-hour pumps, or Intermate infusion systems.<br>• Catheter dressing changes were performed once weekly by the home healthcare nurse.<br>• Patients followed by the ID service were seen for follow-up in the outpatient pediatric ID clinic where potential for transition to oral agents was assessed.	**Key clinical outcomes**<br>• Of 123 OPAT courses with follow-up, 109 (88.6%) resulted in cure, 13 (10.6%) were treatment failures and 1 (0.8%) resulted in OPAT discontinuation because the patient did not have an infection.<br>• The median duration of OPAT was 12 days.<br>• Of 107 courses where OPAT was used for 7 or more days, median duration was 14 days (mode: 12 days, range: 7–51 days). The median duration of OPAT was longest for treatment of hardware infections (38 days), endovascular infections (31 days) and bone and joint infections (20.5 days).<br>• Shorter durations were used to treat intra-abdominal infections (14 days), skin and soft tissue infections (14 days), catheter-related bloodstream infection (12 days), urinary tract infection/pyelonephritis (12 days), complicated pneumonia<br>• Treatment failures occurred in patients with catheter-related bloodstream infections (n = 1), hardware infections (n = 2), intra-abdominal infections (n = 2), central nervous system infection (n = 1), complicated pneumonia (n = 1) and complicated pyelonephritis (n = 1).<br>**Adverse events**<br>• Thirty-six courses (29%) resulted in catheter- or antibiotic-associated complications. Catheter-associated complications (CACs) were less common than drug-associated complications.<br>• CACs included infection, blockage, development of a deep venous thrombosis, dislocation and pain occurred exclusively in patients with PICC lines and those being treated for bone and joint, endovascular and skin and soft tissue infections<br>• Antimicrobial-associated complications occurred most frequently in patients with bone and joint infection, catheter-related bloodstream infection and intra-abdominal infections and in those receiving cefazolin, piperacillin/ tazobactam and vancomycin.<br>• Among 35 patients with complications, 17 (48.6%) had 1 or more unscheduled visits to the emergency department or other healthcare providers, and 8 (22.8%) required rehospitalization	**Financial**<br>• The complications in children receiving OPAT also raise concern about the actual cost-effectiveness and caregiver satisfaction of OPAT in children, which deserves further study	• A retrospective, single<br>• center study over a short time frame.<br>• There were few OPAT patients not followed by the paediatric infectious disease (ID) service, limiting ability to discern differences in outcomes between patients with and without ID involvement.<br>• Definitions of some complications were somewhat subjective (e.g., hepatitis), and may not have prompted all providers to make modifications to antimicrobial therapy.	• Overall cure rate of 89% is slightly lower than previous reported rates between 96% and 99%. These differences may be explained by the fact that study included lengthier follow-up time and included patients with heterogeneous syndromes including difficult to eradicate hardware associated infections.<br>• Opportunities to increase the role of pediatric infectious disease in OPAT initiation and management should be explored<br>• Despite ample literature describing OPAT or PICC line-associated complications many providers initiating and managing OPAT underestimate the associated risks. There is a need to increase provider and patient education about risks associated with OPAT in children.<br>• The complications in children receiving OPAT also raise concern about the actual cost-effectiveness and caregiver satisfaction of OPAT in children, which deserves further study<br>• In this study and others, catheter-related complications were generally seen in children treated for more than 2–3 weeks, suggesting that shortening the duration of OPAT and converting to oral therapy sooner might reduce OPAT complication rates<br>• Opportunities to increase involvement of pediatric ID or antimicrobial stewardship groups in OPAT initiation and management should be explored.
Maraqa et al. (2002)<br><br>USA	Retrospective record review<br><br>179 patients with osteoarticular infections (OAIs); total of 184 episodes.<br><br>Average age was 8.25 years (median 8 years; range 3/12 - 18 years); 100 (54%) were male	**Underlying condition**<br>• No associated conditions reported<br>**Indication for treatment**<br>• OAIs included 116 (63%) acute osteomyelitis, 42 (23%) septic arthritis, and 26 (14%) chronic osteomyelitis.<br>• Among patients with osteomyelitis, 23 (16%) had concomitant septic arthritis	• Not reported	**Setting**<br>• Unclear.<br>**Delivery**<br>• While on OPAT, patients were followed weekly, and a nurse performed weekly laboratory tests looking for OPAT-related complications.<br>• Patients' caregivers had 24-hour access to on-call physicians in both specialties.	**Key clinical outcomes**<br>• OPAT was given through 110 (59.8%) CVLs, 71 (38.6%) peripherally inserted central catheters (PICCs), and 3 (1.6%) peripheral cannulas.<br>• OPAT courses were completed in 118 (64%) OAIs.<br>• Average length of therapy for acute osteomyelitis was 42.2 days. Average length of therapy for septic arthritis was 29.6 days. Patients with chronic osteomyelitis received an average of 58 days of parenteral therapy, followed by oral antibiotic therapy<br>**Adverse events**<br>• Three OPAT courses had both mechanical and infectious complications (2 CVL, 1 PICC).<br>• Infectious complications included 13 cases of local exit-site infection: 10 CVL, 3 PICC, and 4 cases of bacteremia (2 CVL, 2 PICC).<br>• The overall rate of infectious complications was 2.7 per 1,000 catheter-days. The average time to development of infectious complications was 24.5 days.<br>• Non-catheter-related OPAT complications were mostly due to adverse drug reactions (92%) occurring in 60 (32%) courses .<br>• Rehospitalization during OPAT occurred in 48 (26.1%) courses: 28 CVL (26.3%) and 20 PICC (26.7%).	• None reported	• None reported	• Whenever possible, a PICC line should be used since it is safe to use in children<br>• Patient selection for OPAT should take into consideration family readiness and acceptance, the presence of a suitable home environment, as well as issues of reimbursement<br>• An improvement in the quality of life of both the children and their families was reported.<br>• IOPAT had an excellent rate of clinical cure and a low rate of complications for management of childhood osteoarticular infections.<br>• Although oral therapy of OAIs may be preferable, OPAT provides an effective and safe modality of therapy, especially if compliance or bioavailability of oral agents is a concern.<br>• To ensure a good outcome, a team approach is necessary where caregivers are active participants in management and a supportive

					• Rehospitalizations were most commonly due to adverse drug reactions, followed by catheter-related mechanical complications and further surgical management.<br>• Clinical cure occurred in 168 (97.6%) of 172 evaluable OAIs. Twelve (6.5%) courses were nonevaluable due to ongoing therapy or loss to follow-up. Of the four (2.2%) OAIs that failed treatment, one had recurrence and three had persistence of infection			medical team is accessible at all times
**Author, Year Country**	**Design, Sample size, Age of children**	**Underlying condition/ Indication for treatment**	• **Key requirements /influences on offering OPAT/inclusion & exclusion criteria**	**Setting (DTC/Home)/ Delivery**	**Key clinical outcomes/adverse events (Note: for some papers events categorised as adverse by scoping reviewers)**	• **Other outcomes (e.g., finance, family perspectives)**	• **Author-noted limitations**	**Author-noted comments/ recommendations**
Orme et al (2014)<br><br>Australia	An unblinded randomised controlled trial comparing<br><br>Range age: 1.95–16.92<br><br>N=27, 18 inpatients, 19 outpatients	Fever and neutropenia in children receiving chemotherapy for malignancy.	**Inclusion criteria**<br>• Resided within 1 hour travel time (or 30 km radius) with appropriate transport and telephone<br>**Exclusion criteria**<br>• AML or mature B-cell lymphoma<br>• Patients in the induction phase of ALL or receiving predominantly high dose stem cell-supported chemotherapy<br>• History of allergy to penicillins or cephalosporins<br>• families who were non-English speaking.	• Home treatment with Home and Community Care support. After discharge, (HACC nurses visited twice daily to provide general review with vital sign monitoring, cefipime administration and blood tests.<br>• Parents of outpatients recorded tympanic temperature 4–6 hourly. Blood product transfusions (in day procedure centre) and outpatient medical reviews were arranged as deemed appropriate	**Adverse events**<br>There were no adverse events due to outpatient management per se.<br>Antibiotic management was altered for the six outpatients who were readmitted during the course of FN, including change to acyclovir in one patient and addition of or change to amphotericin in two	**Parental**<br>Parent questionnaires show a higher level of QOL for outpatient care on Days 2, 3 and 4 overall, though these differences were not statistically significant.<br>For children, only differences in sleep (7.7 vs. 5.6, P¼0.01) and appetite (6.2 vs. 4.4, P¼0.05) were significant.	• The number of study participants was limited and as such the ability to understand safety, feasibility and risk stratification is limited.<br>• Findings may reflect an overall bias towards an ultra low risk FN subset.<br>• We did not track and record reasons for ineligibility and refusals of patients at initial preconsent when they were identified in oncology clinic.<br>• We did not assess culturally and linguistically diverse families<br>• The majority of families were represented in survey participation by the mother; fathers may have had different views.<br>• Finally, the survey questions chosen were not part of a validated QOL tool	• Further work should aim to examine the effectiveness of outpatient FN management through large cohort studies which may also assess the use of decision tools which are intended to facilitate ambulatory approaches
**Author, Year Country**	**Design, Sample size, Age of children**	**Underlying condition/ Indication for treatment**	**Key requirements /influences on offering OPAT/inclusion & exclusion criteria**	**Setting (DTC/Home)/ Delivery**	**Key clinical outcomes/adverse events (Note: for some papers events categorised as adverse by scoping reviewers)**	**Other outcomes (e.g., finance, family perspectives)**	**Author-noted limitations**	**Author-noted comments/ recommendations**
Reid and Bonadio (2006)<br><br>USA	Retrospective record review (5.5 years)<br><br>29 patients presenting at Emergency Department (ED)<br><br>Age: ranged from 1 to 16 years.	**Underlying condition**<br>• No associated conditions reported<br>**Indication for treatment**<br>• Cellulitis (20), Lobar pneumonia (2), fever without focus (3), pyelonephritis (2), mastoiditis (1), Group A streptococcal tonsillitis (1).	**Inclusion criteria**<br>• Parents had to be considered reliable caretakers<br>• Have access to transportation and a telephone.	**Setting**<br>• Emergency Department.<br>**Delivery**<br>• All patients were instructed to return to the ED within 24 hours for re-evaluation of their condition (follow up ED visit).<br>• Patients with cellulitis had an ink line drawn on their skin to demarcate the outer perimeter of the infection.<br>• Standard practice to instruct parents to inspect the IV site every 4 hours and to seek re-evaluation if there was swelling, redness, or pain of the contiguous skin, or if the IV catheter apparatus became disconnected.	**Key clinical outcomes**<br>• All patients returned for re-evaluation within 24 hours after initial discharge.<br>• 28 patients received a second dose of IV ceftriaxone through the original catheter and were discharged with a prescription for an oral antibiotic.<br>• No patient required hospitalization at the time of follow-up ED visit.<br>• For patients whose cultures subsequently grew a pathogen, none were resistant to ceftriaxone.<br>• No patient experienced adverse event related to antibiotic administration (e.g, rash, diarrhoea, anaphylaxis).<br>**Adverse events**<br>• In one case, a parent removed the IV catheter at home when their child complained of numbness/soreness of the skin at the IV site.	• None reported	• Limited by retrospective design. Although no study patients returned to study hospital after their second visit, it is possible some were treated at other institutions for a deterioration in their condition.<br>• Study population represents a convenience sample so selection bias may have excluded patients who would have made results less compelling.	• Peripheral IV catheters for pediatric patients can be successfully secured and maintained at home by reliable caregivers for a period of 24 hours.<br>• Use of peripheral IV catheters for short-term parenteral therapy for infectious conditions in pediatric outpatients seems feasible.<br>• Because parents were not required to prepare and administer medication at home, the infection control pitfalls inherent to this process were avoided.
Shemesh et al. (1998)	Retrospective analysis (2 years)	**Underlying condition**<br>• Acute lymphocytic leukemia (8), Beta-	**Inclusion criteria**<br>• Patients eligible for immediate discharge on HIAT were	**Setting**<br>• Home<br>**Delivery**	• Patients were examined by their oncologist during home therapy for a mean of one visit per 2.9 days on HIAT (group total, 219 office visits).	**Parental/family**<br>• All 24 parents and patients surveyed were very satisfied	• No pilot study can safely recommend one method of	• Results show that immediate discharge on HIAT is feasible and safe for a preselected

Israel	Retrospective phone survey (re: satisfaction and adverse events) of patients and parents<br><br>30 patients with oncology/ haematology diagnosis; 15 female; 15 male.<br><br>Treated for 60 episodes of fever.<br><br>Median age: 8 years (range 9/12 - 19 years)	thalassemia, splenectomized (4), Wilms' tumor (3), Ewing's sarcoma (2), B-cell lymphoma (2), Rhabdomyosarcoma (2), Other (9<br>**Indication for treatment**<br>• Febrile episode with low risk for septic complications	required to meet the following criteria:<br>• Full parental (and patient, when applicable) consent.<br>• Previous demonstration of proficiency in home care (as judged by the designated nurse).<br>• Less than 1 hour's travelling time from the patient's home to the nearest hospital.<br>• The infection was probably community-acquired (no hospitalization in the previous 48 hours).<br>• No serious comorbidity existed that independently required hospitalization; no signs of shock. 6. The underlying hematologic disease was known and presumably controlled.<br>• No initial evidence of pulmonary or central nervous system infection.<br>• No history of drug or child abuse in the nuclear family.<br>• The patient had a functional indwelling central venous catheter.<br>• Single-parent families, families of low socioeconomic background, and Bedouin families (who live in tents in the desert but have a reliable fresh water source and a refrigerator) were not excluded	• After the central venous catheter was inserted for HIAT, eligible patients and parents received instruction, under close supervision by a designated nurse, on how to administer the fluids or antibiotics and competence evaluated.<br>• Routine follow-up was scheduled 3 to 4 days after initiation of therapy, or earlier if needed.<br>• Parents were actively encouraged to consult the attending oncologist by phone or consult a nurse or the HIAT team on a 24-hour basis.<br>• Patients were instructed to present at the emergency room immediately should any signs of deterioration develop. It was explicitly stated that a parental desire for hospitalization was reason enough for admission.	• A mean of 10.6 days of HIAT were necessary per episode (range, 1 to 24 treatment days), group total 640 days.<br>**Adverse events**<br>• 13 cases (22%) led to hospitalization (unresponsive cases) during or shortly after HIAT.<br>• Of the 42 episodes of fever without an obvious source of infection and neutropenia, eight (19%) eventually led to hospitalization. They all recovered after a mean stay of 8 days in the hospital.<br>• Five out of 12 patients who were not neutropenic were eventually hospitalized (41.6%).<br>• Of the 12 cases of suspected central catheter infection, only two (17%) led to hospitalization.<br>• In all, unresponsive patients were hospitalized for a mean period of 21 days (range, 14 to 36 days). All cases recovered with no obvious sequelae.<br>• Although six patients died during this 2-year period, none of them did so within 3 months of HIAT, and all deaths were clearly associated with disease progression.<br>• Central catheter infections developed in 2 patients following HIAT. These patients were hospitalized, but it was not necessary to remove the catheters.<br>• No serious adverse drug reactions were observed during HIAT.<br>• Forty cases of fever were initially treated with ceftriaxone. Nine of these (23%) eventually led to hospitalization, four for Pseudomonas infections. Four of six cases who received a combination of ceftazidime and vancomycin (67%) were eventually hospitalized, two because of resistant Pseudomonas infection and two because of resistant catheter infection. Of the 10 cases who received ceftazidime alone, none required hospitalization. None of the splenectomized cases, who received cefotaxime, needed hospitalization.	with home IV antibiotic therapy (HIAT) and wanted to use it at the next hypothetical episode.<br>• Five of the 24 felt only partially competent at the first episode and needed expert help.<br>• No one noted a specific adverse drug reaction, but five patients cited fatigue as a common problem during therapy.<br>• None encountered serious mechanical problems.<br>• Parental satisfaction was almost complete. Some parents indicated they were a bit uncomfortable and insecure on the first days of home treatment.<br>• Experience suggests that it might prove useful to send a nurse for one home visit during the first days of treatment.	treatment over another.<br>•	immune-compromised pediatric population.<br>• A randomized, controlled, prospective trial is needed to reach valid conclusions on home therapy with hospitalization
**Author, Year Country**	**Design, Sample size, Age of children**	**Underlying condition / Indication for treatment**	**Key requirements /influences on offering OPAT/inclusion & exclusion criteria**	**Setting (DTC/Home)/ Delivery**	**Key clinical outcomes/adverse events (Note: for some papers events categorised as adverse by scoping reviewers)**	**Other outcomes (e.g., finance, family perspectives)**	**Author-noted limitations**	**Author-noted comments/ recommendations**
van der Laag & van de Weg (1995)<br><br>The Netherlands	Research design unclear (no methodology noted); appears to be a retrospective record review (2.5 years).<br><br>15 patients at this hospital 33 intravenous antibiotic courses at home<br><br>The 15 home-treated patients included 7 children from the Wilhelmina Children's Hospital (age range 7-17 years)..	**Underlying condition**<br>• Cystic fibrosis<br>**Indication for treatment**<br>• Exacerbations of chronic respiratory infections due to aeruginosa	**Inclusion criteria**<br>• The inclusion criteria for training sessions are that the patient should: have had at least one intravenous antibiotic course in hospital within the last year; not have any complication of the disease for which hospitalization is necessary be compliant with all other kinds of treatment (physiotherapy, good nutrition, etc.)<br>• Insurance companies agreed to pay for leasing an electronic infusion pump and elastomeric balloon pumps.<br>• In-hospital testing of allergic reactions to drugs, the organization of home care and final instructions to patients and parents	**Setting**<br>• Home<br>**Delivery**<br>• Two cystic fibrosis (CF)nurses were appointed to inform and instruct patients and their families about all procedures concerning antibiotic treatment.<br>• After a successful training a contract between the patient, parents and hospital was signed, in which the hospital agreed that home care was appropriate and guaranteed readmission in the case that home care failed or complications occurred.<br>• The specialized CF nurse was authorised to give intravenous injections and was responsible for the instruction of patients and parents and control at home during the treatment period.<br>• Prior to discharge home, patients treated with	**Key clinical outcomes**<br>• In all cases of home treatment patients improved their clinical condition, lung function and general well-being compared to former courses in hospital.<br>• The home-treated children all needed frequent intravenous antibiotic treatment.<br>• Most of the other children received only one course in a year; four of them have been trained for home treatment and will probably have this next time<br>**Adverse events**<br>• Four instances of complications during the home treatment periods for which a 24 h on-call service existed. These were: technical problems with the electronic pump, air bubbles in the cassettes of the pump due to the ceftazidime solution, and phlebitis for which a new venous access was needed (two cases).	**Parental**<br>• Parents, especially of those in the younger age group (< 12 years), felt reluctant to accept such a great responsibility for their child's treatment. Mothers were also anxious that the family and other siblings would suffer under the stress of home care. Hospital admissions were seen to give some kind of relief to the family unit.<br>• In the older age group (12-18 years) the young people missed their daily contact with hospital staff and fellow patients on the ward but were glad to be able to attend their own school and to be at home without disruption of family life.<br>• After a successful home-treatment period, patients agreed eagerly to proposals for home care or even requested it on new admission for exacerbation of infections.<br>• Indications for home care had to be considered carefully, since 10 cases showed so much anxiety after a former period of home treatment that a further course	• None reported	• In a specialized disease like CFs home treatment with antibiotics seems feasible for a restricted number of patients.<br>• Complications of the disease and anxiety of patients and parents, even if they have had adequate training and instruction, are the most important reasons for not starting home treatment.<br>• Patients younger than 8 years are less suitable, as they do not need intravenous treatment very often and stress at home may be too great to have successful home treatment.<br>• During the information and instruction phase of home care attention has to be paid not only to the technical aspects of infusion pumps and drug delivery, but also to the social and psychological issues, to enable the full effect of antibiotic treatment at home to be felt.

				ceftazidime continuously in combination with tobramycin twice daily (doses adjusted after measuring top and trough levels).		was given completely in hospital instead of at home		
Van Winkle et al. (2008)<br><br>USA	Retrospective record review (2 years 9 months)<br><br>34 patients<br><br>The mean age for children in the group was 4.4 years (range, 12 days–12 years), with 56% male and 44% female. The age distribution was relatively even between 2 weeks and 12 years of age.	**Underlying condition**<br>• 2 patients had leukaemia, 2 had cystic fibrosis, no other associated conditions reported.<br>**Indication for treatment**<br>• 30 patients had infectious disease (e.g., osteomyelitis, septic arthritis, pyelonephritis, resolving sepsis related to bacteraemia, cellulitis, appendicitis, mastoiditis).<br>• 2 patients were undergoing treatment for leukaemia<br>• 2 treated for exacerbations of cystic fibrosis	• No detail reported apart from, discharge catheter care was provided by the Kaiser Orange County home health agency, which is directly affiliated with the treatment center. Between the home health care agency and the medical center, there was no standardized approach regarding this care. The care was provider dependent.	**Setting**<br>• Home<br>**Delivery**<br>• Between the home health care agency and the medical center, there was no standardized approach regarding this care.<br>• The care was provider dependent.	**Key clinical outcomes**<br>• 33 of 34 patients completed therapy as an outpatient (97%).<br>• 39 PICCs were placed in the 34 patients who were treated with outpatient parenteral antibiotic therapy and included in this study.<br>**Adverse events**<br>• 13 of the 39 PICCs (33.3%) had a complication requiring removal of the catheter. One child was readmitted due to accidental displacement at home. 5 children were close to the end of therapy and completed it with oral antibiotics. The remaining 7 PICCs were replaced as follows: 5 with a second PICC to continue outpatient therapy, and 2 with peripheral IV catheters to complete a brief duration of outpatient therapy.<br>• None of the children changed to oral antibiotic therapy had treatment failure requiring need for subsequent IV antibiotics.<br>• There were no incidences of phlebitis or suspected or confirmed catheter infection or sepsis.<br>• There was a general trend toward increased odds of complications with PICCs placed midline (odds ratio 12.95; 95% CI: 0.95–175.40; P 0.0540).	**Financial**<br>• A financial analysis was completed on the cost of home health versus the projected cost of hospitalization for the same period of time. The average daily cost for home health treatment was $115 compared with the average daily inpatient cost of $1185.<br>• Total cost savings on a subset of 26 patient episodes analyzed was $504,858, which was $19,418 per patient episode or $1070 per day of home health care for the 472 potential inpatient days analyzed.	• Small sample size.<br>• Results may be skewed as larger centers more likely to be treating more complex patients who may be more prone to complications.	• OPAT has a significant cost savings over the alternative of inpatient treatment.<br>• OPAT has a positive effect on hospital utilization, which is a critical factor in smaller paediatric wards where bed availability and the difficulty of transfer to referral hospitals are issues.
**Author, Year Country**	**Design, Sample size, Age of children**	**Underlying condition / Indication for treatment**	**Key requirements /influences on offering OPAT/inclusion & exclusion criteria**	**Setting (DTC/Home)/ Delivery**	**Key clinical outcomes/adverse events (Note: for some papers events categorised as adverse by scoping reviewers)**	**Other outcomes (e.g., finance, family perspectives)**	**Author-noted limitations**	**Author-noted comments/ recommendations**
Wierniko wski et al (1991)<br><br>Canada	Pilot programme (research design unclear)<br><br>13 children<br><br>Age not reported<br><br>and their families successfully completed courses of intravenous antibiotic therapy at home. Seven of these children were treated on more than one occasion, giving a total of 22 episodes.	**Underlying condition**<br>• Cancer<br>**Indication for treatment**<br>• Febrile neutropenia	**Inclusion criteria**<br>• Children who had indwelling right atrial catheters were eligible for the pilot program if they met the following criteria: (a) child afebrile for at least 48 hours, after starting intravenous antibiotics in hospital; (b) child on appropriate antibiotic(s), as determined from culture results; (c) child clinically stable in the opinion of the attending hematologist; and (d) parent(s) interested, motivated, and able to administer intravenous antibiotics as assessed by the pediatric oncology nurse.	**Setting**<br>• Home<br>**Delivery**<br>• Child's parent was instructed by a pediatric oncology nurse on the process of antibiotic administration, including aseptic technique, infusion procedure, catheter care, and troubleshooting.<br>• Parents were required to give their child's antibiotic(s) in hospital under supervision prior to discharge, so that their proficiency at performing this task could be assessed by the pediatric oncology nurse.<br>• The pediatric clinical pharmacist provided the parents with information on drug reconstitution, dosage, and side effects, and arranged for the supply of antibiotics.<br>• Each family was sent home with enough antibiotics and supplies to complete a 10-14-day course of therapy as appropriate.<br>• A follow-up appointment was made	**Key clinical outcomes**<br>• The children who received home therapy spent an average of 3 days in hospital followed by an average of 10 days at home. By comparison, the average length of stay for hospital treatment of febrile neutropenic episodes was 12 days during the same 6-month period<br>• All episodes of fever and neutropenia were treated successfully with home IV antibiotic therapy.<br>• No reported adverse drug reactions, drug toxicity, or catheter complications such as occlusion, but some minor problems were encountered, such as shortages of some supplies. In every case, the shortages were discovered far enough ahead that additional supplies were obtained for the parent within 24 h.	**Parental**<br>• Two families met the criteria for entry into the pilot program, were instructed, but decided, prior to discharge, not to proceed and the children remained in hospital for antibiotic treatment.<br>• The feedback from the parents who participated in this program was uniformly positive.<br>**Financial**<br>• Cost analysis hampered by inability to cost inpatient treatment accurately. Average estimated daily cost of a "hospital bed" (all types was $618; therefore, estimated cost of 12 days of inpatient therapy at $7,416 (noted to probably overestimates the true cost of such treatment).<br>• Using hospital acquisition costs, a somewhat clearer estimate of the cost of home therapy estimated at $2,781. These do not include the costs that are borne directly by the families of the children.<br>• The Regional Home Care program paid for the drugs and supplies that the families needed for home therapy during our pilot study. When the families had third-party coverage, the cost of the drugs was borne by the insurer	• Not reported	• Home IV antibiotic therapy appears a safe and efficacious alternative to hospital management of children with malignant diseases admitted with fever and neutropenia.<br>• A cost analysis of the program indicates that home therapy is considerably cheaper than in-hospital treatment.<br>• However, costs for parents/families are expensive; especially if the parent must forego wages she/he would otherwise have earned in order to stay home with the child to administer antibiotics<br>• Home IV antibiotic program for children with malignant disease was a positive experience for the children and the families involved.

				to the out-patient clinic usually within 1 week of discharge.<br>• In addition, 24-h on-call coverage was available to all families-a service we provided already. If the child developed a fever at any time while on home therapy, the family was instructed to bring the child back to hospital for re-evaluation.				
